# Ectopic ovarian pregnancy after intracytoplasmic sperm injection with testicular spermatozoa - a case report

**DOI:** 10.5935/1518-0557.20170029

**Published:** 2017

**Authors:** Javier García-Ferreyra, Roly Hilario, Julio Dueñas

**Affiliations:** 1 FERTILAB Assisted Reproduction Laboratories, Lima, Peru; 2 PROCREAR Fertility Center, Lima, Peru

**Keywords:** ectopic pregnancy, ovarian pregnancy, ART

## Abstract

Ovarian ectopic pregnancy is a rare event in both natural and assisted human reproduction settings. There are few reports of this event after *in vitro* fertilization. Diagnosis can be challenging, since it requires specific medical expertise. Patients with this condition call for careful management during treatment so as to not affect their fertility potential. This paper describes the case of a woman submitted to ICSI and embryo transfer who subsequently had an ovarian ectopic pregnancy and underwent a laparoscopic partial right oophorectomy

## INTRODUCTION

Assisted reproduction technologies (ART) have helped infertile couples achieve satisfactory implantation and pregnancy rates. Nevertheless, ART procedures have their inherent risks, one of which is ectopic pregnancy (EP). In cases of ectopic pregnancy, the blastocyst implants outside the uterine cavity. The incidence of EP ranges between 1.2-1.4% for spontaneous pregnancies ( Rana *et al.*, 2013), and from 1.5% to 2.1% in patients undergoing IVF/ICSI (Londra *et al.*, 2015). Approximately 98% of EP cases occur in the fallopian tubes, one percent in the abdominal region (Atrash *et al.*, 1987), and, in extremely rare occasions, in the ovaries, with an incidence of 0.5-3% (Raziel *et al.*, 2004). An ectopic pregnancy involves direct fertilization of an unreleased mature egg inside an ovary, or retrograde migration of the embryo into an ovary via the fallopian tube. Normally, during embryo transfer (ET), the embryos are placed 1.5-2.0cm from the uterine fundus. Ovarian pregnancy is thought to occur due to a retrograde migration of an embryo via the tube and the implantation of such embryo in the ovary. This report describes a case of ovarian pregnancy after ICSI with testicular spermatozoa and fresh embryo transfer.

## CASE REPORT

A 25-year-old female and her 57-year-old male partner came to our fertility center on September 2016 for an ICSI procedure with cryopreserved testicular spermatozoa. The patient had regular menstrual cycles, and normal uterus, tubes and ovaries. FSH, LH and estradiol levels at day 3 were 5.25IU, 5.67IU, and 30.08pmol/l, respectively. The ovarian cycle was stimulated using gonadotropins according to previously established stimulation protocols (Tavmergen *et al.*, 2002). Starting on day 2, the patient received a daily dose of 187.5IU of rFSH (Gonal^®^, Merck Serono Laboratories, Peru) until day 11, when ovulation was triggered by 250µg of recombinant human chorionic gonadotropin (Ovidrel^®^, Merck Sereno Laboratories, Peru). A total of 10 oocytes were collected, and nine were injected with testicular sperm from her partner, leading to the development of four blastocysts on day 5. Two blastocysts were transferred and the remaining ones were frozen. Micronized Progesterone (600mg/day, vaginally; Geslutin^®^, Tecnofarma, Peru) was used for luteal support. Two weeks later, tests showed a BhCG level of 44.69mIU/ ml; eight days later, the patient's BhCG level had increased to 9963mIU/ml. At seven weeks, the patient reported moderate abdominal pain. Transvaginal ultrasound examination did not find intrauterine pregnancy, but a paraovarian mass in her right ovary. The patient was hospitalized and underwent laparoscopic examination, and was diagnosed with right ovarian ectopic pregnancy. Blood and tissue were removed from the pelvic area and a partial right oophorectomy was performed. Histopathology confirmed the diagnosis of ovarian pregnancy with one sac. The patient recovered well from surgery.

## DISCUSSION

It has been estimated that 10% of the women hospitalized for ectopic pregnancy in the developing world die (Leke *et al.*, 2004). Several authors have reported that ART procedures increase ectopic pregnancy rates (Marcus & Brinsden, 1995; Strandell *et al.*, 1999; Clayton *et al.*, 2006), and associations have been described between in vitro fertilization (IVF) and a 2-5% risk of EP, which may increase in the presence of tubal disease (Clayton *et al.*, 2006). According to Marcus & Brinsden (1995), 4.5-6% of all cases of extrauterine pregnancy and 0.35% of all clinical pregnancies are ovarian pregnancies. Choi *et al.* (2011) looked into 3081 cases of ectopic pregnancy and found that 49 were cases of ovarian pregnancy (1.59%), as similarly reported by Grimes *et al.* (1983); Gaudoin *et al.* (1996) and Raziel *et al.* (2004).

Retrograde migration of the blastocyst into the fallopian tube and implantation in an ovary may be one of the causes of ovarian EP. Ovarian pregnancy is diagnosed based on four criteria: (1) the fallopian tubes and the fimbriae must be intact and separated from the ovary; (2) the pregnancy must occupy the normal position in the ovary; (3) the ovary must be attached to the uterus through the utero-ovarian ligament; and (4) there must be ovarian tissue attached to the pregnancy in the specimen (Spiegelberg, 1878). However, diagnosis is difficult and relies on the suspicion and judgment of experienced physicians, since this rare condition is often asymptomatic until the ovaries are ruptured.

Possible risk factors to ovarian ectopic pregnancy include use of intrauterine devices, history of laparotomy or laparoscopic surgery, endometriosis, ART procedures, and uterine anomalies. In our report, the patient was healthy and had no history of surgery or infectious/inflammatory process. Oophorectomy or salpingo-oophorectomy is the traditional treatment for ovarian ectopic pregnancy. However, a wedge resection or the partial removal of the gestational product alone is the ideal treatment to maintain fertility, as shown in this study. Finally, diagnosis of EP is of the utmost importance, as it may lead to proper surgical treatment to safeguard and preserve fertility.
